# Characterising microbial protein test substances and establishing their equivalence with plant-produced proteins for use in risk assessments of transgenic crops

**DOI:** 10.1007/s11248-012-9658-3

**Published:** 2012-10-12

**Authors:** Alan Raybould, Peter Kilby, Gerson Graser

**Affiliations:** 1 Syngenta, Jealott’s Hill International Research Centre, Bracknell, Berkshire, RG42 6EY UK; 2 Syngenta Biotechnology Inc., 3054 E. Cornwallis Rd, PO Box 12257, Durham, NC 27709 USA

**Keywords:** Risk assessment, Surrogate protein, Bioactivity, Intactness, Post-translational modification

## Abstract

Most commercial transgenic crops are genetically engineered to produce new proteins. Studies to assess the risks to human and animal health, and to the environment, from the use of these crops require grams of the transgenic proteins. It is often extremely difficult to produce sufficient purified transgenic protein from the crop. Nevertheless, ample protein of acceptable purity may be produced by over-expressing the protein in microbes such as *Escherichia coli*. When using microbial proteins in a study for risk assessment, it is essential that their suitability as surrogates for the plant-produced transgenic proteins is established; that is, the proteins are equivalent for the purposes of the study. Equivalence does not imply that the plant and microbial proteins are identical, but that the microbial protein is sufficiently similar biochemically and functionally to the plant protein such that studies using the microbial protein provide reliable information for risk assessment of the transgenic crop. Equivalence is a judgement based on a weight of evidence from comparisons of relevant properties of the microbial and plant proteins, including activity, molecular weight, amino acid sequence, glycosylation and immuno-reactivity. We describe a typical set of methods used to compare proteins in regulatory risk assessments for transgenic crops, and discuss how risk assessors may use comparisons of proteins to judge equivalence.

## Introduction

Most transgenic crops for commercial use have been genetically engineered to produce new proteins. Among other things, the proteins may improve the crop’s resistance to insect attack, confer tolerance to various herbicides, make the crop more nutritious, improve its processing properties, or act as markers to identify the crop. Risk assessments for the consumption or cultivation of such crops use studies that test relevant properties of the novel protein to predict the likelihood that the crops will harm human health or the environment (Hérouet et al. 2005; Craig et al. 2008; Romeis et al. 2008; Sanvido et al. 2012).

Many studies for risk assessment require grams of highly (≥90 %) pure protein. Often it is not possible to prepare the required amount of purified protein from transgenic plants because the proteins are produced in low amounts and their purification from the plant matrix is technically extremely difficult, if not impossible (Hérouet et al. 2005). It is, however, relatively easy to produce sufficient protein of acceptable purity by over-expressing the protein in fermentable microbes, such as *Escherichia coli*. Microbial proteins can be purified from disrupted bacterial cells using standard methods, including precipitation and chromatography. After purification, the protein is concentrated, desalted and, in most cases, lyophilised. The resulting powder is a microbial test substance, which is used to measure properties of the protein considered relevant for assessing risks.

For non-pesticidal proteins, the requirement for large amounts of protein is mainly due to mammalian toxicity studies. A typical study for evaluation of acute oral toxicity (e.g., based on the US Environmental Protection Agency guideline OCSPP (formerly OPPTS) 870.1100 requires a minimum of 5 male and 5 female mice each to be given a single dose of at least 2,000 mg protein per kg body weight. Depending on the weight of the animals, this study alone can use about 2 g of protein. Sometimes, a repeated-dose oral toxicity study is used (e.g., based on OECD guideline 407). Such studies require a minimum of 5 male and 5 female mice each to be given a single dose of at least 1,000 mg protein per kg body weight daily for 28 days, which may require over 25 g of active protein (Delaney et al. 2008).

In addition to mammalian toxicology studies, regulations require pesticidal proteins to undergo ecotoxicology testing, and some of the studies may use large amounts of protein. A typical study of acute oral toxicity in birds (e.g., based on US EPA guideline OCSPP 850.2100) requires a minimum of 5 male and 5 female birds (usually bobwhite quails) each to be given a single dose of at least 2,000 mg protein per kg body weight, representing over 2 g of protein. Studies of honey bee brood (Oomen et al. 1992) may expose each of 3 hives to 1 L of sucrose solution containing the protein at 10 times the concentration in the pollen of the transgenic crop. If the pollen contains 50 μg protein per g fresh weight, and the density of sucrose solution is 1.2 g/ml, 1.8 g of protein would be needed for the study.

High purity of the protein test substance aids interpretation of the results of studies. If adverse effects are observed in a toxicology or ecotoxicology study, one needs to be confident that the effects are caused by the protein and not by an impurity in the test substance. In some ecotoxicology studies, it is possible to expose animals to high concentrations of protein via diets containing tissue from the transgenic crop. Statistically significant differences between groups of organisms fed material from a transgenic crop and groups fed similar non-transgenic crop tissue are common (e.g., Wandeler et al. 2002; Obrist et al. 2006; Faria et al. 2007; Rosi-Marshall et al. 2007; Bøhn et al. 2010). Interpretation of such results regarding effects of the transgenic protein is difficult because the transgenic and non-transgenic crops differ by more than just the presence or absence of the transgene, and therefore the test materials almost certainly differ by more than just the presence or absence of the protein coded by that transgene (e.g., Parrott 2008).

While there are clear advantages of microbial test substances for regulatory studies, it is essential that the substances are properly characterised. Their purity must be estimated so that the correct amount of test substance is used to give the required amount of protein in a given study. Information about solubility is crucial as many studies require aqueous solutions of the test substance. Failure of the test substance to dissolve or remain in solution could invalidate a study. It is also crucial to show that the protein in the microbial test substance is a suitable surrogate for the protein produced by the transgenic crop, because there may be intended or unintended differences between the microbial and plant proteins. Corroboration of the hypothesis of no significant difference between the microbial and plant proteins in relevant properties is taken as evidence that the proteins are functionally and biochemically equivalent for the purposes of studies that inform risk assessments for the transgenic crop.

This paper provides an overview characterisation and equivalence^1^ studies for microbial test substances that support their use in risk assessment studies as surrogates for plant-expressed proteins. Its purpose is to show the variety of data that is produced in order to judge the robustness and applicability to transgenic crop risk assessments of studies that use microbial test substances. The paper does not provide exhaustive detail about experimental design, nor does it provide a complete set of data for a single test substance, such as provided by Fuchs et al. (1993a, b), Gao et al. (2004, 2006b) and Hérouet et al. (2005). Instead, it concentrates on general principles, summaries of current methods, potential problems and interpretation of multiple lines of evidence to provide an up-to-date review of current practice in establishing the suitability of microbial proteins to act as surrogates for plant-produced transgenic proteins.

As discussed below, a microbial test substance need not be identical to the plant protein for which it is a surrogate. Equivalence means that the microbial and plant proteins are deemed sufficiently similar for the purposes of specific studies that contribute data for risk assessments. An important corollary of this definition is that it is not feasible to devise a procedure that will determine equivalence for all test substances for all uses. The methods described below should be regarded as options for a risk assessor to build a weight of evidence to judge whether or not an individual test substance is suitable for a particular use. They are not a series of tests that trigger an objective “pass” or “fail” decision based on universal criteria that distinguish equivalence from non-equivalence.

## Methods for characterising test substances

### Solubility

Determination of the solubility of a protein test substance in aqueous solutions is essential for its further characterisation because most analytical techniques require the substance in a solubilised form. The solubility determination of a protein test substance is therefore commonly the first experiment conducted during test substance characterisation. Furthermore, the solubility determination provides important information about the possible delivery vehicles in animal toxicity studies and non-target organism effects studies.

The solubility of microbial test substances in water and other aqueous solutions can be determined by a simple optical test. Defined volumes of the solvent are added to the lyophilised test substance and its solubility—the highest concentration at which the test substance is dissolved completely—is determined by visual inspection, or confirmed by analytical methods of total protein determination described below, or both. For toxicology studies, solubility of the test substance in water is desirable because it eliminates the possibility of side effects from other components of the solvent, such as buffers. Unfortunately, many protein test substances have limited solubility in water, meaning that buffers such as tris(hydroxymethyl)methylamine (TRIS), *N*-cyclohexyl-3-aminopropanesulfonic acid (CAPS), or various phosphate buffers are often required to dissolve test substances. Other additives, such as ethylenediaminetetraacetic acid (EDTA) dithiothreitol (DDT) or Tween20 may also be needed to stabilise the protein in the solution. For some studies, such as acute oral toxicity in mice, a homogenous suspension of test substance may be an acceptable alternative to a solution.

In studies that require aqueous solutions of the protein test substance, the best buffer is the simplest one that maintains the test substance in solution for the period of use. Where buffers are used, it may be necessary to determine whether they significantly affect the results. In non-target organism effects studies, for example, preliminary experiments to determine the effect of the buffer on the test species are advisable, and inclusion of control groups exposed to buffers in effects studies should be considered (Romeis et al. 2011). Sometimes it is possible to modify study designs to cope with test substances that are difficult to dissolve. The effect of proteins on the development of honeybee brood may be determined by exposing hives to an aqueous solution of sucrose in which the test substance is dissolved. The sucrose solution is placed in feeders near the hive, and worker bees carry solution back to the hive and feed it to the developing brood (Oomen et al. 1992). For test substances that are easily maintained in solution, the required amount of protein could be delivered to a hive in a single 1L batch of treated sucrose solution placed near the hive at the beginning of the experiment. For test substances that are difficult to keep in solution over a longer period, each hive could be exposed to 200 ml of treated solution on each of the first 5 days of the study.

### Purity

**Fig. 1:**
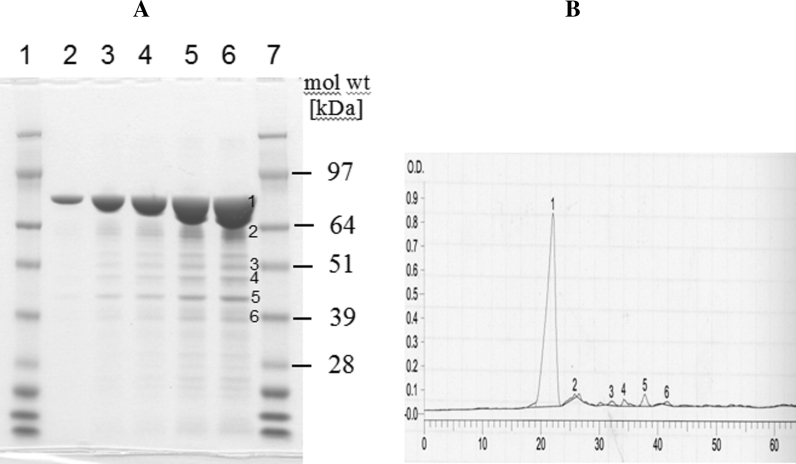
Coomassie *Blue* stained SDS-PAGE gel of a microbial produced Vip3Aa19 test substance analyzed by densitometry. **a** Coomassie stained SDS-PAGE gel. *Lanes* *1* and *7* Molecular weight standard SeeBlue^®^Plus2 (Invitrogen; bands indicated as kDa); *lanes 2*, *3*, *4*, *5* and *6* 0.3, 0.6, 1.2, 2.4, 4.8 μg protein test substance. The molecular weight of Vip3Aa19 corresponds to ca. 89 kDa. **b** Densitometric analysis of the Coomassie stained SDS-PAGE gel using a laser densitometer. The signals derived from the individual protein bands are translated into* peak areas* (indicated by the numbers on the gel and on the densitometry graph). The* peak areas* signal can be used to calculate the percentage of each protein within the total protein fraction of the test substance. The analysis showed that the protein comprising the protein of interest (Vip3Aa19) represented 91.4 % of the total protein fraction in the test substance. (Color figure online)

The purity of a microbial test substance is usually determined in two stages. First, the proportion of the test substance that is protein is determined using standard laboratory methods such as BCA™ (bicinchoninic acid) (Hill and Straka 1988; Walker 1996), Bradford analyses (Bradford 1976), or, in cases of highly pure (>90 %) test substance preparations, spectrophotometrically by measuring absorbance at 280 nm (Gill and von Hippel 1989). Secondly, the proportion of protein that is the protein of interest (POI) is determined by sodium dodecyl sulphate polyacrylamide gel electrophoresis (SDS-PAGE) of the protein test substance, followed by staining with Coomassie Blue and quantitative densitometry (Fishbein 1971). The proportion of total protein comprising the POI is calculated as the area under the peak representing that protein divided by the total area under all peaks. The purity of the test substance is simply calculated as the proportion of the test substance comprising protein multiplied by the proportion of protein comprising the POI. Figure 1 shows a typical densitometric analysis of a Coomassie Blue stained SDS-PAGE gel.

The purity of a protein test substance is reported as per cent POI weight-by-weight. The purity of the protein test substance provides information for further analysis of the protein test substance, and for accurate dosing of the POI in mammalian toxicity and non-target organism effects studies.

Highly pure test substances (>90 % POI) are preferred, because they reduce the probability of adverse effects arising from impurities such as proteins from the *E. coli* expression system (e.g., Franken et al. 2000). High purity test substances also allow the highest possible dosing of the POI at a minimal volume to animals by gavage where limit doses are required for acute toxicity exposure studies in rodents.

## Measures of test substance equivalence

### Intactness and immuno-reactivity

Analysis of the molecular weight of the microbial and plant proteins provides information on whether they have been truncated or degraded in a sample; therefore, molecular weight is commonly called a measure of intactness. Molecular weight determination can also detect modifications of proteins, such as glycosylation, and insertions or deletions of amino acids. Immuno-reactivity refers to the ability of a protein to bind specific antibodies. Loss of immuno-reactivity may indicate modifications to a protein that change its biochemical or functional properties.

Western blot analysis, also known as protein immuno-blotting, is a convenient method for comparing the intactness and immuno-reactivity of protein samples. In western blotting, proteins are separated by SDS-PAGE and transferred from the gel to a membrane in a second electrophoresis step called “blotting” (Burnette 1981). The proteins are immobilised on the membrane, which thereby acquires an exact copy of the original protein gel image. Once blotted, the POI can be detected using specific antibodies, allowing the POI to be identified in complex mixtures such as protein crude extracts from plants. Western blot analysis displays the apparent molecular weight of the POI by comparing its electrophoretic mobility with that of a molecular weight standard. Kurien and Scofield (2006) provide a recent review of western blotting techniques.

Western blotting is a powerful technique for analysis of the immuno-reactivity and intactness of proteins of interest from different matrices. Side-by-side comparison of the apparent molecular weight of the proteins from different sources provides compelling evidence for equivalence because major differences in modification of the proteins would result in changes in mobility. Confirmation of the intactness of a protein within its matrix also supports the reliability of associated ELISA analyses, as breakdown of the protein could lead one to over-estimate its concentration.

**Fig. 2:**
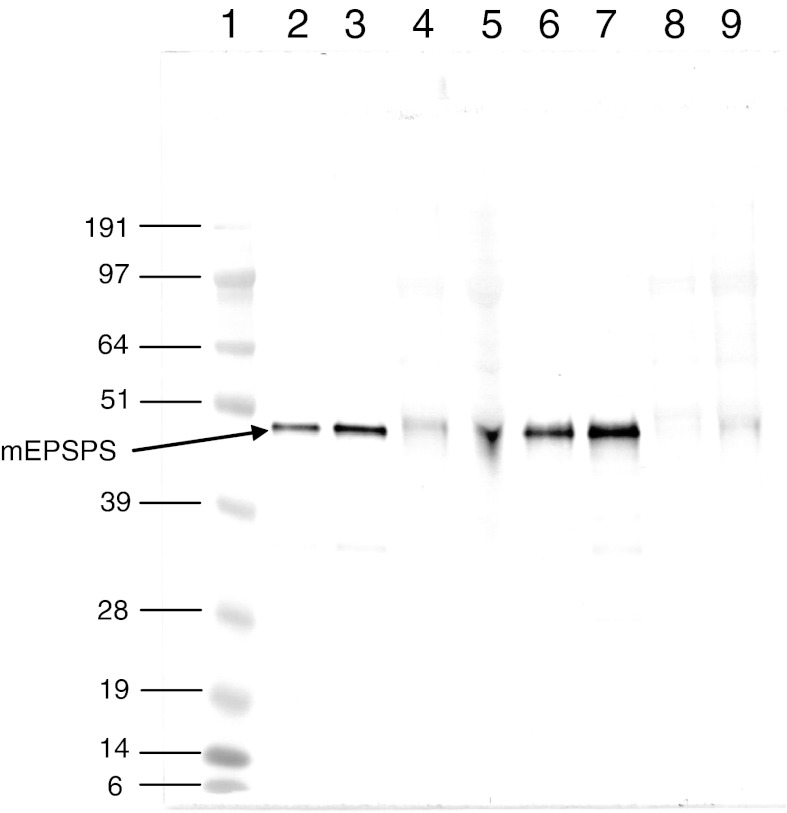
Western blot analysis of mEPSPS from recombinant *E. coli* and from transgenic maize. *Lane* *1* Molecular weight standard SeeBlue^®^Plus2 (Invitrogen; bands indicated as kDa); *lanes* *2* and *3* 7.5 and 15 ng mEPSPS microbial mEPSPS, respectively; *lanes* *4* and *5* 7.5 and 15 ng mEPSPS from GA21 maize (crude extract), respectively; *lanes* *6* and *7* 7.5 and 15 ng mEPSPS from GA21 maize (purified using immunoaffinity chromatography), respectively; *lanes* *8* and *9* 3.5 and 6.9 μg total protein from non-transgenic maize, respectively. The molecular weight of mEPSPS corresponds to about 47.4 kDa

An example of western blotting to compare the intactness and immuno-reactivity of a microbial and plant POI is shown in Fig. 2. The analysis shows that mEPSPS derived from recombinant *E. coli* and from GA21 maize bind rabbit anti-EPSPS polyclonal antibodies, and have the same apparent molecular weight. The loss of resolution observed for the mEPSPS protein bands derived from the maize crude extract is explained by the interference from large amounts of protein derived from the plant matrix. The endogenous maize EPSPS in the negative control (non-transgenic) maize extract appears as a faint band because the antibody is not able to discriminate between the native maize EPSPS and mEPSPS.

### Intact mass

The determination of the molecular weight of a protein by western blots is relatively imprecise (Sadeghi et al. 2003). Exact estimates of intact protein masses can be obtained by mass spectrometry (MS). Two MS methods can be used to determine the intact mass of both microbial and plant proteins: electrospray MS, often implemented on a quadrupole-time-of-flight (Q-TOF) type mass spectrometer, and Matrix Assisted Laser Desorbtion Ionisation (MALDI) MS on a MALDI-TOF instrument (Sundqvist et al. 2007).

For MS analysis of microbial proteins, Q-TOF analysis is preferred because it achieves higher mass accuracy than MALDI-TOF analysis (Sundqvist et al. 2007). Q-TOF machines are able to distinguish between proteins with single amino acid substitutions, or other low molecular weight modifications, such as methionine oxidation. Such differences would not be detected by MALDI-TOF MS. Plant-produced POIs can in principle be analysed by either Q-TOF or MALDI MS. MALDI is currently the method of choice because its greater sensitivity enables analysis of small amounts of plant POIs that are difficult to obtain in large, pure batches (Hérouet et al. 2005).

Obtaining the precise mass of the POI provides direct evidence about the form of the protein present in the transgenic crop, and can make a strong case for sequence identity with the microbial protein; for example, one may be able to demonstrate that the plant protein is processed in a particular way, whether or not it has a leader sequence, or that it has not been unexpectedly glycosylated. No other method can provide such detail about the chemical form of the intact protein within the plant.

Analysis of the intact mass of plant-produced POIs by MS presents significant problems. First, sufficient POI must be isolated from the plant and concentrated into a small volume (a few μL). Secondly, the POI must be of high purity, so that peaks from contaminating proteins or other compounds do not confound the analysis. In general, these problems are reduced the higher the concentration of the POI in the transgenic plant. Thirdly, the isolation method must not modify the mass of the POI. Polyphenol oxidation during extraction is a particular problem (Le Bourvellec and Renard 2012) and methods to reduce it, such as including in extraction buffers compounds that adsorb phenols (e.g., Loomis and Battalie 1966), may be necessary.

### Protein sequence

The amino acid sequence of a protein provides useful information about its likely structure and function (e.g., Eisenhaber et al. 1995); therefore, amino acid sequence comparisons are conducted as tests of the biochemical and functional equivalence of microbial and plant proteins. Two methods are used routinely: N-terminal sequencing and peptide mass mapping.

N-terminal sequences of both microbial and plant-produced POIs can be determined using Edman sequencing (Edman and Begg 1967). The POI is converted to a phenylthiocarbamyl protein by reaction of the N-terminal amino acid with phenylisothiocyanate. The modified N-terminal amino acid is released by cleavage with trifluoroacetic acid and converted to a phenylthiohydantoin, which can be identified after separation by chromatography or electrophoresis. The amount of sequence obtainable is limited because the conversion reactions do not go to completion. However, the sequence of the first 10 amino acid residues can almost always be determined for a microbial protein, and where sequence can be obtained for plant protein the comparison is straightforward. Edman data are semi-quantitative and are able to detect mixed N-terminal forms should they be present in plant or microbial protein samples. Many plant POIs are N-terminally modified into forms that are blocked to chemical sequencing, most commonly by acetylation (Martinez et al. 2008). In these circumstances, N-terminal sequence comparison is not possible and MS provides an alternative approach to confirm the N-terminal amino acid sequence for the plant POI; however, technical hurdles, as indicated below, limit its application in many cases.

Peptide mass mapping is the application of MS to the characterisation of plant and microbial protein sequences. Outside plant biotechnology, the term normally refers to the application of MALDI-peptide mass fingerprinting methods for protein identification (Jensen et al. 1997; Ren et al. 2005; Dauly et al. 2006). Protein identification is considered reliable if the coverage is at least 15 % of the sequence and 5 or more peptides are matched (Jensen et al. 1997).

Peptide mass mapping has been used to sequence proteins for regulatory submissions for transgenic crops; for example, Gao et al. (2006a) used MALDI-TOF peptide mass fingerprinting to characterise Cry1A.105 and Cry2Ab produced in MON 89034 maize. Each protein was separated on an SDS-PAGE gel and then digested with trypsin (Williams et al. 1997). The masses of the tryptic fragment peptides were measured using MALDI-TOF MS (Billeci and Stults 1993) and were compared with those of the predicted peptides from the expected amino acid sequence of the respective proteins. Where a mass matched that of a predicted peptide, the sequence of the peptide was considered assigned as the expected sequence. The method matched 52 peptides, confirming 43.8 % (516 of 1,177 amino acids) for full-length Cry1A.105, and matched 32 peptides, confirming 44.4 % (283 of 637 amino acids) for full-length Cry2Ab2.

Gao et al. (2004) took a similar approach to the characterisation of Cry34Ab1 and Cry35Ab1 expressed in *Pseudomonas* and transgenic maize, and to Cry1F produced in *Pseudomonas* and transgenic cotton (Gao et al. 2006b). Scott et al. (2006) used the same gel tryptic digest method but combined it with single ion monitoring on an electrospray single quadrupole instrument to compare 2mEPSPS produced in *E*. *coli* and in GHB614 cotton; peptides from the microbial 2mEPSPS were identified in the cotton 2mEPSPS with a coverage of over 90 % and the calculated masses for the peptides were identical.

**Fig. 3:**
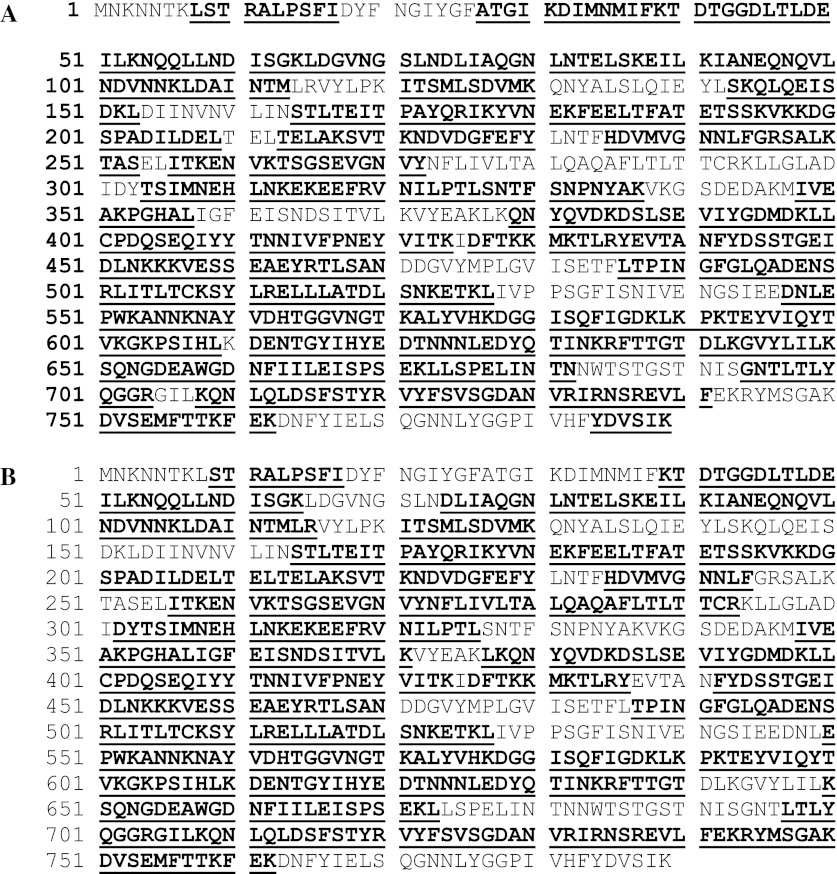
Amino acid sequence coverage map for **a** the microbial Vip3Aa19 and **b** for the plant-derived Vip3Aa19. The sequence* highlighted* and *underlined* represents peptides identified. Evidence for 75.2 % of the sequence was obtained by combining the results of analyses using three separate enzymes

Another approach to identification of tryptic fragments from SDS-PAGE gels uses nano-liquid chromatography-MS/MS (LC–MS/MS) conducted on a Q-TOF instrument (Marvin et al. 2000). Peptide mass mapping MS/MS data are interrogated with the Mascot search tool (Perkins et al. 1999) using a database containing the predicted amino acid sequence of the plant or microbial protein. Data from individual peptides confirm parts of the sequence and together build up a coverage map for the whole protein. Analysis of the microbial protein is conducted in the same way and the two coverage maps used to confirm the presence of the same protein in both plant and microbial samples. Figure 3 shows the maps for microbial and plant-derived Vip3Aa19 with 75 % and 71 % coverage of the proteins respectively.

Obtaining MS/MS spectra of peptides at high mass accuracy enables confirmation of the identity of individual peptides from the protein digest. Each MS/MS spectrum contains information about the amino acid sequence within the peptide, which is not provided by the other methods. This provides two advantages: first, protein identity is verified without the need to achieve high levels of sequence coverage; and secondly, peptides from contaminating proteins can be easily shown to not be associated with the protein of interest. In some cases, the N-terminal peptide can also be covered, which provides reliable data regarding the amino acid sequence and would make the separate N-terminal sequence analysis indicated above redundant.

No threshold value for percentage of sequence coverage of a protein obtained from nano-LC–MS/MS data has been established; however, in the proteomics field it is common to accept the identification of proteins in a sample where high quality spectra from only two peptides unique to the protein of interest have been recorded (Bradshaw et al. 2006). Indeed more recently the need for even the second peptide has been questioned (Gupta and Pevzner 2009).

It is common in peptide mass mapping that the coverage for the microbial and plant proteins is not identical. This does not necessarily imply a difference in sequence between the two proteins. For example in the case of the data for Vip3Aa19 shown in Fig. 3 coverage of individual peptides and percentage coverage is similar but not identical. This might have occurred for a number of reasons. The samples might not have been the same strength and commonly the plant protein is the weaker sample showing as in this case lower percentage coverage. In LC–MS/MS, the process by which the mass spectrometer selects ions for fragmentation is to some extent random and the same peptides are not selected for fragmentation in each run even in comparable runs of an identical sample (Liu et al. 2004; Elias et al. 2005).

### Glycosylation

Over half of the proteins in plants are estimated to be glycosylated (Apweiler et al. 1999). Glycosylation typically consists of the addition of complex structures derived from carbohydrates. Glycosylation can alter the physiochemical properties of a protein, such as its tolerance of heat, functional activity, protein folding, transport and half-life (Solá et al. 2007). *N*-glycosylation is a common glycosylation motif in plant proteins (Strasser et al. 2004; Nagels et al. 2012). The sequence motif Asn-Xxx-Ser/Thr, or in some rare cases Asn-Xxx-Cys, where Xxx is any amino acid except Pro, is required for *N*-glycosylation. The absence of these sequences can therefore completely exclude *N*-glycosylation of the protein. For other glycosylation types, programs have been developed using algorithms to predict glycosylation sides with over 90 % accuracy (Hamby and Hirst 2008), and provide useful information regarding the glycosylation potential of a protein.

Glycosylation has been much studied in connection with potential increased allergenicity (Wilson et al. 2001). However, increasing knowledge of plant glycosylation has recently led to the conclusion that carbohydrate moieties are probably insignificant as clinically important allergen determinants (Altmann 2007). Nevertheless, plant proteins are analysed for glycosylation status in order to detect possible changes in function as part of protein equivalence assessments. Transgenic proteins in plants are not intended to be glycosylated and recombinant proteins produced in *E. coli* are not glycosylated (e.g., Baneyx and Mujacic 2004); therefore, demonstrating the absence of glycosylation adds to the weight of evidence that the POI and the microbial protein are functionally equivalent. Differences in glycosylation status might be regarded seriously owing to potential variation in physicochemical properties of the proteins. The analysis of glycosylation in protein equivalence studies is routinely accomplished using immuno-blot assays. Proteins are separated by gel electrophoresis and electro-transferred to a membrane as in western blot analysis. Once immobilised on the membrane, glycosyl-residues are detected using antibodies or sensitive chemical methods (Haselbeck and Hösel 1990; Westermeier and Marouga 2005) so that only glycosylated proteins result in visible bands.

**Fig. 4:**
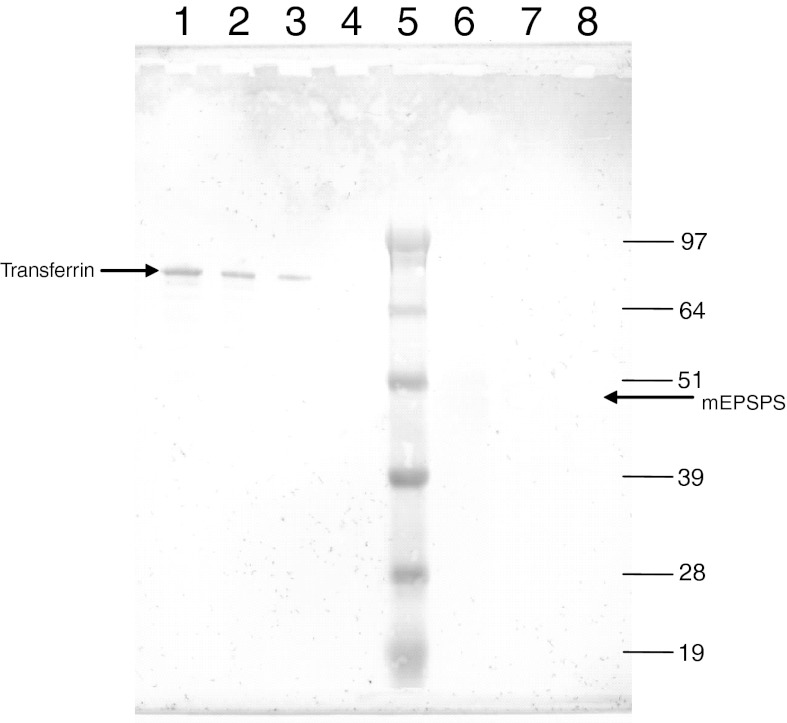
Glycosylation analysis of mEPSPS from recombinant *E. coli* and transgenic GA21 maize. *Lanes* *1*, *2* and *3* 100, 50, 25 ng, transferrin (positive control, molecular weight of ca. 80,000 and contains ca. 5 % glycan moieties by weight), respectively; *lane* *4* 2,000 ng creatinase (a nonglycosylated protein used as negative control); *lane* *5* molecular weight standard SeeBlue^®^Plus2 (Invitrogen; bands indicated as kDa); *lane* *6* 732 ng microbial produced mEPSPS protein; *lanes* *7* and *8* 732 and 1,463 ng plant produced mEPSPS protein. The expected molecular weight of mEPSPS is indicated by the *arrow* on the *right* side of the gel

Figure 4 shows an immuno-blot glycosylation analysis of mEPSPS derived from recombinant *E. coli* and from extracts of leaf material from transgenic GA21 maize. Transferrin, a protein known to be glycosylated, was used as a positive control, and creatinase, a protein known to be non-glycosylated, was used as a negative control. The control proteins were used to confirm the integrity of the assay and to establish its sensitivity. In this analysis, visualisation of glycosylated proteins was achieved by chemical oxidation of glycan moieties, which were then covalently labelled with digoxigenin (DIG), and detected with an alkaline phosphatase-linked antibody sensitive to DIG. Alkaline phosphatase catalyzed a colorimetric reaction resulting in stained bands representing glycosylated proteins. Loading different amounts of the positive control allowed the sensitivity of the assay to be estimated. The results indicate that both mEPSPS proteins are not glycosylated, or that glycan moieties occur at a frequency of less than one glucose equivalent per molecule of mEPSPS.

Further evidence about glycosylation status may be obtained from the western blot analysis comparing the plant-derived protein with the microbial test substance. Typical plant *N*-glycosylation patterns are rather complex and increase the mass from between 1 and 2 kDa per glycosylation site (Wilson et al. 2001). Such increases in the total mass of the plant protein would be evident from a side-by-side comparison with the microbial protein on a western blot.

### Activity

Many transgenic proteins are enzymes. For these proteins, it is important to test the hypothesis that the activity of the protein in the microbial test substance is equivalent to that of the protein produced in the transgenic crop. Similarity in activity is a good predictor of similar biological interactions, such as mammalian toxicity and effects on non-target organisms.

Enzyme activity assays vary depending on the chemistry of the reaction catalyzed. Specific activity is reported in units per amount of enzyme. Units are defined for each enzymatic reaction in terms of the product produced, or the substrate used, over time under defined conditions. To calculate specific activity, the concentration of enzyme within the activity assay must be estimated. This is done routinely by enzyme-linked immunosorbent assay (ELISA) (Tijssen 1985), a well-established method to quantify proteins in different matrices. Often ELISA cannot distinguish between active and inactive proteins, which can result in inaccurate estimates of specific activity. Hence activity studies are often conducted on crude plant extracts to avoid inactivation during purification of enzymes produced by the transgenic plant. Another consideration is that the plant matrix may reduce the specific activity of enzymes because of the action of proteases and reactive secondary metabolites, such as phenols, and other effects such as rapid pH changes during extraction. The best comparison of specific activity of microbial and plant-produced proteins may therefore be between a crude extract from a transgenic plant and microbial test substance spiked into extract from a suitable non-transgenic control plant.

The reduced activity of enzymes in some plant matrices is illustrated by an example of transgenic maize resistant to herbicides containing glyphosate. Glyphosate inhibits 5-enylpyruvylshikimate-3-phosphate synthase (EPSPS), an enzyme in the biochemical pathway that synthesises aromatic amino acids from shikimic acid (Amrhein et al. 1980). Expression of a modified EPSPS (mEPSPS), containing two amino acid substitutions, provides glyphosate resistance in Event GA21 maize owing to reduced binding of glyphosate to the modified enzyme (Dill 2005). The activities of mEPSPS derived from recombinant *E. coli* and from transgenic maize were determined using a EPSPS-specific activity assay based on the detection of orthophosphate released during the transfer of the enolpyruvate moiety of phosphoenolpyruvate to shikimate 3-phosphate (Stalker et al. 1985). The released phosphate is detected by its forming a complex with Malachite Green and molybdate under acid conditions, which is detected by spectrophotometry at 660 nm (Itaya and Ui 1966; Lanzetta et al. 1979).

The microbial mEPSPS had about 9 times the specific activity of the plant enzyme (Table 1). However, when the microbial enzyme was added to an extract from non-transgenic maize, the specific activity of the microbial enzyme was only about twice that of the plant enzyme, indicating that the maize extract inhibits mEPSPS activity. This is important information when judging whether the microbial mEPSPS is a suitable surrogate for the plant-produced EPSPS.

**Table 1:** Comparison of the specific activity of mEPSPS from various sources

Sample	Mean specific activity (Units/mg mEPSPS) ± SD
Microbial mEPSPS	6,700 ± 32
GA21 maize mEPSPS	734 ± 16
Microbial mEPSPS + non-transgenic maize extract	1,560 ± 21

One unit of mEPSPS activity is defined as the release of 1 nmol of phosphate per minute under standard assay conditions (Padgette et al. 1987)

The activity of pesticidal proteins is measured as the concentration or dose of the toxin that affects a given proportion of individuals of a test organism in a certain time. For insecticidal proteins, such “bioactivity” is usually reported as an LC_50_, the concentration of the toxin that kills 50 % of a sensitive test insect a certain time after exposure to the toxin. While enzyme activity assays should be reproducible within ranges established during the assay validation, insect bioassays are expected to have greater within- and among-assay variability than enzyme assays in their absolute responses owing to biological variation among the individual insects tested (Robertson et al. 1995). To minimise extraneous variation, tests of biological equivalence of insecticidal proteins from plant and microbial sources, bioactivity should be estimated concurrently under uniform conditions using individuals from the same cohort of insect larvae randomly allocated to treatments (Romeis et al. 2011).

Bioactivity is usually assessed using a target pest of one of the transgenic events that the equivalence study will support. If the target pest is difficult to rear in the laboratory, or shows variable responses to a protein, a non-target pest species may provide a more rigorous test of the hypothesis of no difference in bioactivity; for example, the target pest of Cry3Bb1 produced in MON863 maize is western corn rootworm (*Diabrotica vergifera vergifera*), but Colorado potato beetle (CPB; *Leptinotarsa decemlineata*) is preferred for bioassays of Cry3Bb1 because of its greater sensitivity to the protein (e.g., Duan et al. 2008).

Comparisons of bioactivity may be particularly useful when the microbial protein is known to differ from the plant protein in amino acid sequence because of point mutations in the transgene that occurred during or after plant transformation. An example is Vip3A, an insecticidal protein isolated from the vegetative cells of *Bacillus thuringiensis*. Vip3Ais toxic to several lepidopterous pests of maize and cotton (Lee et al. 2003), and provides control of these pests when produced in transgenic crops. Vip3A produced in VipCot cotton (Kurtz et al. 2007) and Pacha maize (Dively 2005) is a 789 amino acid protein denoted Vip3Aa19. Vip3A produced in MIR162 maize is also 789 amino acids long, but differs from Vip3Aa19 by one amino acid: isoleucine instead of methionine at position 129. The protein in MIR162 maize is denoted Vip3Aa20. The change from methionine to isoleucine at position 129 is a conservative substitution. Both amino acids are uncharged, nonpolar and have similar molecular weights (149 vs 131); thus, the difference in amino acids is unlikely to change the three dimensional structure of the protein. An additional reason for expecting similar properties of Vip3Aa19 and Vip3Aa20 is that the amino acid difference occurs outside the protein tryptic core (Lee et al. 2003).

Studies using microbial test substances containing Vip3Aa19 have been used to support risk assessments for MIR162 maize (Raybould and Vlachos 2011). In surface incorporation bioassays using first-instar *Spodoptera frugiperda* (Fall armyworm), the 120-hour LC_50_ estimated from 3 independent bioassays was 137 ng Vip3A/cm^2^ (95 % CI = 82–199) for microbial Vip3Aa19, and 154 ng Vip3A/cm^2^ (95 % CI = 94–222) for Vip3Aa20 from MIR162 maize. The similar bioactivity was part of the justification for using Vip3Aa19 studies to support risk assessments for MIR162 maize (US EPA 2009).

Corroboration of the hypothesis that the microbial and plant test substances do not have different activities is important for interpreting the results of toxicity and ecotoxicology studies. If the activity of the microbial protein is no different from that of the plant protein, then the dose or concentration of microbial protein used in a study can be compared directly with predicted environmental concentrations of proteins that may result from cultivation of the transgenic crop. Suppose that several representative surrogate non-target organisms are exposed to diets containing a microbial protein at 500 μg/g diet with no observable adverse effects, and that predicted highest exposures of non-target organisms to the protein via cultivation of the transgenic crop are no greater than 50 μg/g diet. Provided that dietary exposure in the test diet is confirmed, it follows that one can infer with high confidence that the no observable adverse effect concentration (NOAEC) of the protein to all species represented by the tested surrogates is greater than or equal to their highest exposure in the field (the worst-case expected environmental concentration or EEC). In risk assessments this may be presented as a hazard quotient (HQ): EEC/NOAEC ≤0.1 (Raybould et al. 2011). This would be strong corroboration of the hypothesis that non-target organisms will not be exposed to harmful concentrations of the protein via cultivation of the transgenic crop (e.g., Raybould et al. 2007).

If the hypothesis that the microbial and plant proteins do not have different activity were rejected, it would not necessarily mean that the microbial test substance is unsuitable for risk assessment studies. If other studies show that the proteins are equivalent apart from activity, the difference in activity could be allowed for in risk assessments. In the example above, if the microbial protein were found to have half the activity of the plant protein, that is the LC_50_ of the microbial protein is twice that of the plant protein, then one could correct the estimate of the NOAEC to half the value based on the concentration of the protein the microbial test substance. After correction for bioactivity, the HQs would be ≤0.2—still strong corroboration of the hypothesis of no harm to non-target organisms, but giving a little less confidence in a conclusion of negligible risk compared with studies done with a test substance of equivalent activity to the plant protein. Finally, one could argue that greater potency of the protein in the microbial test substance may allow a higher NOAEC to be set for risk assessments of the transgenic crop containing the less potent protein; however, in practice this is unlikely to be convincing as lower activity of the plant protein may be due to effects of the plant matrix or to inactivation during purification, not to intrinsically higher activity of the microbial protein.

To date, the desired traits of most commercial transgenic crops are based on the production of proteins that are enzymes or toxins. Methods to measure the activity of these proteins are conceptually straightforward. New traits may be based on proteins that are not so simple to assay for activity. One method of increasing water-use efficiency of maize is the production of a cold-shock protein derived from *Bacillus subtilis* (CSPB). CSPB binds to single-stranded DNA or RNA. Its binding activity may be revealed in vitro by fluorescence from a labelled double-stranded probe as it becomes opened by the protein (Castiglioni et al. 2008). This assay has been used to determine the equivalence of a microbial and plant-produced CSPB (Pester et al. 2009). Water-use efficiency may also be improved by the production of new transcription factors (e.g., Kasuga et al. 1999). There are in vitro methods for determining the specificity and affinity of transcription factors (Jolma and Taipale 2011). These methods could fulfil the role of functional assays when assessing test substance equivalence in cases where activity assays as described above are not applicable.

## Judging the equivalence of proteins

Microbial and plant proteins may differ in several ways: the differences may be unintended results of changes during transformation or test substance production, or may be intended to assist production of the test substance; and the differences may be single amino acid substitutions, additions of short amino acid tags, or large deletions of parts of the microbial or plant proteins.

Unintended differences from the plant protein may arise during production of microbial protein and these differences tend to be minor. One common source of variation is cleavage of the N-terminal methionine when proteins are produced in microbes and its retention in plant-produced proteins. Another source of variation is mutations in the gene for the POI during transformation of the plant or the microbial expression vector; mutations tend to result in differences of one or two amino acid residues between the plant and microbial proteins (e.g., Raybould and Vlachos 2011).

Occasionally, the microbial protein is designed to be different from the plant protein. Short tags of 6-10 amino acids such as histidine may be added to the N- or C-terminus of the microbial protein to aid purification (Schmitt et al. 1993). Sometimes, the protein produced in the plant may be hard to produce in microbes. For example, many insect-resistant transgenic crops produce truncated (or “activated”) forms of Cry proteins. Producing similarly truncated Cry proteins as microbial test substances is often difficult. The solubility of Cry proteins varies depending on the organism in which they are produced (Khasdan et al. 2003), and although truncated Cry proteins are soluble in plants, they are often insoluble in microbes. In such cases, one option is to produce a full-length microbial protein and truncate it by treatment with a protease such as trypsin (e.g., Porcar et al. 2010). Alternatively, it may be possible to use the full-length protein in, for example, non-target organism effects studies if the proteins are equivalent in attributes other than length.

The suitability of a microbial protein for a risk assessment study depends on whether it is determined to be equivalent to the plant protein in properties relevant to the purpose of the study. Usually, a weight-of-evidence approach is taken; that is, no single study determines whether or not the proteins are equivalent, and equivalence is determined by evaluation of the results of several studies such as those described above and outlined in Table 2. Other lines of evidence, such as whether for single amino acid differences both amino acids are neutral or acid, or both are hydrophilic or hydrophobic, and whether the substitution has occurred in part of the protein known to determine important properties such as bioactivity, may also be considered.

**Table 2:** Examples of tests to establish equivalence between plant proteins and microbially produced protein surrogates

Parameter compared	Method	Contribution to equivalence assessment	Interpretation of results
Intactness	Western blot analysis	Detection of potential amino acid sequence differences owing to insertions, truncations or degradation; detection of modifications such as glycosylation	Insertions, truncations or modifications indicate potential differences in physicochemical properties including functional activity. Differences may be acceptable depending on results of other parameters identified and the purpose of the safety study
Immuno-reactivity	Western blot analysis	Detection of potential differences in immuno-reactivity	Differences in binding to specific antibodies indicate differences in protein structure; further tests should be conducted to judge impact on equivalence
Intact mass	Mass spectrometry	Detection of insertions, truncations, substitutions and other modifications with higher accuracy and precision than western blotting	As for intactness
Protein sequence	N-terminal sequencing; mass spectrometry	Detection of potential differences in amino acid sequence	Amino acid sequence contributes to the structure and function of a protein. Differences in sequence may be acceptable depending on results of activity assays and the purpose of the safety study
Glycosylation	Immuno-blot analysis	Detection of potential differences in glycosylation status	Glycosylation affects many properties of proteins including stability and function. It has been claimed that glycosylation affects the allergenicity of proteins, although recent work casts doubt on this suggestion. Nevertheless, differences in glycosylation status might be regarded seriously owing to potential variation in physicochemical properties of the proteins
Functional activity	Enzymatic activity assay, insecticidal bioassay	Detection of potential differences in specific catalytic activity (enzymes) or insecticidal bioactivity (toxins)	Confirmation of equivalent activities confirms equivalent protein folding (tertiary and quaternary structure). Depending on the results of other equivalence tests, differences in activity may be acceptable. Differences in activity may be allowed for in safety studies. For example, margins of exposure could be based on comparisons of activity, not concentration

Equivalence is judged separately for each test substance based on a weight of evidence

It is important to realise that equivalence does not imply that the plant and microbial proteins are identical. Equivalence it is intended to mean that the microbial protein is sufficiently similar biochemically and functionally to the plant protein such that studies using the microbial protein provide reliable information for risk assessment of the transgenic plant. “Sufficiently similar” cannot be defined completely objectively, but is a judgement by risk assessors about whether studies using microbial protein provide reliable and robust tests of risk hypotheses that the cultivation of the transgenic plant will not cause harm. Decisions about the suitability of a particular microbial protein should therefore concentrate on properties that predict harm, and it follows that the microbial protein could be deemed sufficiently similar to the plant protein for some studies but not for others. Those features that are most important should receive the most attention, depending on the intended use of the test substance. For example, equivalent bioactivity may be most important for non-target organism studies, similar glycosylation and immuno-reactivity may be the main requirements for allergenicity studies, while analysis of amino acid sequence may be best for determining suitability for studies that compare enzymatic degradation of proteins.

## Conclusions

Safety studies using purified microbial proteins may provide important data to assess the risks to human and animal health and to the environment from the use of transgenic crops (Garcia-Alonso et al. 2006; Delaney et al. 2008; Romeis et al. 2008). The studies are carried out to internationally accepted guidelines that specify factors such as replication, test duration, measurement endpoints and validity criteria, to maintain the repeatability and reliability of the studies (Delaney et al. 2008; Romeis et al. 2011).

The usefulness of a study with microbial protein does not depend solely on the experimental design elements noted above. It is also essential that the microbial test substance is a suitable surrogate for the plant-produced transgenic protein for the purposes of the study. Suitability as a surrogate does not imply that the microbial and plant proteins must be identical, only that relevant properties of the microbial test substance and the plant protein are sufficiently similar, such that studies with the microbial protein reliably predict the probability of harmful effects that may result from human, animal or environmental exposures to the protein via the transgenic crop. Variation in the functions of proteins, the purposes of studies, and opinions of decision-makers about the relevance of particular differences between proteins, means that it is not feasible to define one set of equivalence criteria that applies to all test substances for all uses. The suitability of test substances as surrogates must be judged individually based on a weight of evidence from studies comparing the microbial and plant proteins for properties including activity, molecular weight, amino acid sequence, glycosylation and immuno-reactivity. Establishment of the suitability of the test substance, along with experimental designs that follow international guidelines, will ensure that studies with microbial protein provide reliable information about the risks posed by the use of transgenic crops.

## Footnotes

1 Studies may be conducted to test whether a protein produced in one transgenic event is functionally and biochemically equivalent to the protein produced in a second transgenic event. These are often termed “bridging studies”, but are identical to the equivalence studies described here.
